# Predictors of Individual Response to Placebo or Tadalafil 5mg among Men with Lower Urinary Tract Symptoms Secondary to Benign Prostatic Hyperplasia: An Integrated Clinical Data Mining Analysis

**DOI:** 10.1371/journal.pone.0135484

**Published:** 2015-08-18

**Authors:** Ferdinando Fusco, Gianluca D’Anzeo, Carsten Henneges, Andrea Rossi, Hartwig Büttner, J. Curtis Nickel

**Affiliations:** 1 Urologic Clinic, University Federico II of Naples, Naples, Italy; 2 Eli Lilly Italy S.p.A., Sesto Fiorentino, FI, Italy; 3 Lilly Deutschland GmbH, Bad Homburg, Germany; 4 Department of Urology, Queen’s University, Kingston, Ontario, Canada; Oklahoma University Health Sciences Center, UNITED STATES

## Abstract

**Background:**

A significant percentage of patients with lower urinary tract symptoms (LUTS) secondary to benign prostatic hyperplasia (BPH) achieve clinically meaningful improvement when receiving placebo or tadalafil 5mg once daily. However, individual patient characteristics associated with treatment response are unknown.

**Methods:**

This integrated clinical data mining analysis was designed to identify factors associated with a clinically meaningful response to placebo or tadalafil 5mg once daily in an individual patient with LUTS-BPH. Analyses were performed on pooled data from four randomized, placebo-controlled, double-blind, clinical studies, including about 1,500 patients, from which 107 baseline characteristics were selected and 8 response criteria. The split set evaluation method (1,000 repeats) was used to estimate prediction accuracy, with the database randomly split into training and test subsets. Logistic Regression (LR), Decision Tree (DT), Support Vector Machine (SVM) and Random Forest (RF) models were then generated on the training subset and used to predict response in the test subset. Prediction models were generated for placebo and tadalafil 5mg once daily Receiver Operating Curve (ROC) analysis was used to select optimal prediction models lying on the ROC surface.

**Findings:**

International Prostate Symptom Score (IPSS) baseline group (mild/moderate vs. severe) for active treatment and placebo achieved the highest combined sensitivity and specificity of 70% and ~50% for all analyses, respectively. This was below the sensitivity and specificity threshold of 80% that would enable reliable allocation of an individual patient to either the responder or non-responder group

**Conclusions:**

This extensive clinical data mining study in LUTS-BPH did not identify baseline clinical or demographic characteristics that were sufficiently predictive of an individual patient response to placebo or once daily tadalafil 5mg. However, the study reaffirms the efficacy of tadalalfil 5mg once daily in the treatment of LUTS-BPH in the majority of patients and the importance of evaluating individual patient need in selecting the most appropriate treatment.

## Introduction

Lower urinary tract symptoms (LUTS) secondary to benign prostatic hyperplasia (BPH) are a common problem, affecting more than 50% of men aged 50 years and older [1].

Medical treatment has focused mainly on the use of α-blocking agents and 5-α reductase inhibitors, either alone or in combination, and aims to alleviate symptoms as well as alter the course of disease progression and prevent complications [2]. Treatment options for LUTS-BPH have since increased with regulatory approval of tadalafil 5mg once daily, a long-acting phosphodiesterase type 5 (PDE-5) inhibitor, initially in the US in 2011 and subsequently in the EU and other major territories in 2012 [3]. Treatment of LUTS-BPH, either alone or with coexisting erectile dysfunction (ED), with PDE-5 inhibitors and notably tadalafil 5mg, has recently been added to EU-wide treatment guidelines for non-neurogenic LUTS [4].

The efficacy of once daily tadalafil 5mg in LUTS-BPH has been demonstrated in four randomized controlled trials (RCTs) [5; 6; 7; 8]. At a lower dose of 2.5mg per day, tadalafil did not consistently alleviate symptoms of LUTS-BPH while higher doses of 10 and 20mg per day provided only minimal additional improvement over the 5mg once daily dose [5]. Assessment of treatment response (primary endpoint) was based primarily on the International Prostate Symptom Score (IPSS), a validated, self-administered, 1-month recall questionnaire that has good reliability for recall of obstructive and urinary problems and their global impact on quality of life (QoL). The IPSS is the most widely used instrument to assess the severity of BPH-related LUTS-symptoms and gauge response to treatment [9; 10].

An integrated analysis of the four RCTs confirmed that tadalafil 5mg achieved significantly greater improvements in total IPSS score, IPSS voiding subscore, IPSS storage subscore and IPSS QoL Index score versus placebo [11]. A separate analysis of IPSS storage and voiding subscores, showed both were significantly improved in the active treatment arms compared with placebo (p<0.001) and that both storage and voiding subscores made a nearly linear contribution to total IPSS in a 4:6 ratio that was maintained from baseline to endpoint [12].

In pooled subgroup analyses, significant improvements in IPSS total score were observed regardless of baseline LUTS severity (IPSS <20/≥20), age (≤65/>65 years), recent use of α-blocking agents or PDE-5 inhibitors, total testosterone level (<300/≥300ng/dl), or prostate-specific antigen (PSA) predicted prostate volume (≤40/>40ml), while tadalafil was well tolerated across all subgroups [13].

A further post-hoc integrated analysis of the data from the four RCTs showed that approximately two-thirds of tadalafil-treated patients achieved a clinically meaningful improvement (CMI) in LUTS-BPH symptoms, as defined by a total IPSS improvement of ≥3 points or ≥25% from randomization to endpoint at Week 12 [14]. Moreover, tadalafil 5mg once daily, demonstrated increasing benefit over placebo as the efficacy threshold was raised from ≥25% to a demanding ≥50% and ≥75% improvement in IPSS [14].

Being able to identify which individual patient is most likely to respond well to treatment with placebo or tadalafil, rather than just knowing its average benefit to a subgroup of patients, would be clinically useful and consistent with the growing trend towards more patient-tailored treatment [15]. Treatment directed at patients most likely to achieve CMI would help address the problem that for too many patients with LUTS-BPH, medical therapy achieves only a fair-to-good improvement in symptoms [16].

In this integrated clinical data mining analysis, we set out to identify the factors associated with response to placebo or tadalafil 5mg once daily in an individual patient with LUTS-BPH. Implicit in a study of this nature was the need to carefully estimate the true prediction performance of a factor for unknown patients.

## Methods

### Study design

This clinical data mining analysis was based on the Knowledge Discovery in Databases (KDD) process and was set up to be consistent with the underlying principles of data mining [17].

Applied data mining algorithms were considered suitable only if a graphical presentation could be obtained that could be followed by practicing physicians. We therefore focused on models that were easily visualised or those expected to yield good predictive outcomes. Our aim was to produce an output that could be displayed on paper and used by clinicians and so we decided at the outset to adopt the simplest model first. This can be seen by the inclusion of single decision rules (SDRs). These models consider just one clinical variable at a time to predict one response variable, without any additions, and they perform well.

Rigorous care was taken to evaluate the prediction error for unknown data. Every effort was made to control for potential data mining biases (i.e. those induced by applying too flexible data mining algorithms or those stemming from the desire to achieve 100% accurate predictions). To this end we adhered to a pre-specified statistical analysis plan (SAP), which did not allow for removal of data points. We set out our experience first, wrote down our approach, and kept to it without deviation. We did not intend to optimize prediction performance further than what had been pre-specified. To do so would only bias results for models that are adapted and optimized for a specific combination for the training algorithm and evaluation method, and which are thereby unlikely to capture the clinical information that is predictive in clinical practice.

More extensive methodological details not covered here are provided in Supporting Information.

### Data sources and pre-processing

**Fig 1:**
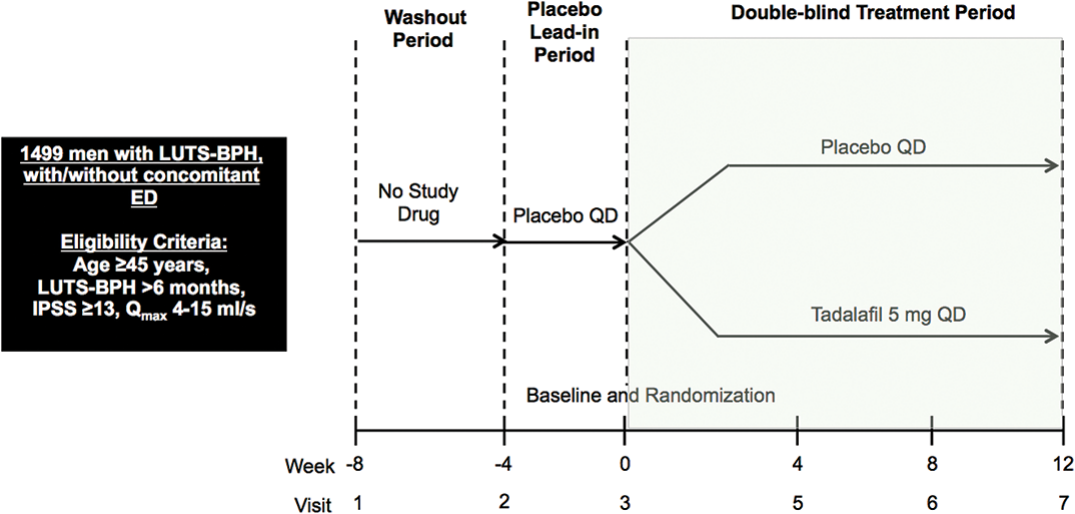
Design of the four randomised, placebo-controlled trials of tadalafil 5mg once daily in patients with LUTS-BPH.

Data for this clinical data mining analysis were pooled from four, randomized, placebo-controlled clinical studies (NCT00384930, NCT00827242, NCT00855582, NCT00970632), all of which had a broadly similar design and enrolled patients with LUTS-BPH (Fig 1) [6; 7; 8; 16]. Common inclusion criteria for all four studies were age ≥45 years, LUTS-BPH duration of >6 months, total IPSS ≥13, and maximum urinary flow rate (Qmax) ≥4 to ≤15ml/s prior to the placebo lead-in period. Patients were excluded if PSA was >10ng/ml (or for PSA 4–10ng/ml, prostate malignancy had to be excluded), if post-void residual (PVR) urine volume was ≥300ml, or if they had used finasteride or dutasteride within 3 or 6 months (12 months in one study), respectively. Following screening and, if needed, a washout period for LUTS-BPH or ED medications, patients entered a 4 week placebo lead-in period. On completion, patients were randomized to study treatment with tadalafil 5mg once daily for 12 weeks. Minor differences between the studies included the following: one enrolled patients with BPH and concomitant ED [7]; one was a dose-finding study in which tadalafil was administered at doses of 2.5mg, 5mg, 10mg, 20mg once daily [16]; one included a tadalafil 2.5mg treatment arm [7]; and one included an additional tamsulosin 0.4mg treatment arm [8].

For the purposes of this clinical data mining analysis the study population (N = 1,499) consisted solely of subjects in the intent-to-treat (ITT) population who had been allocated to tadalafil 5mg once daily or placebo irrespective of an IPSS baseline assessment (Table 1). Data from the tadalafil 2.5mg, 10mg and 20mg once daily treatment groups did not form part of the data mining analysis, as these doses are not approved for the treatment of LUTS-BPH.

**Table 1:** Baseline characteristics of the ITT population used for data mining analysis.

Characteristics*	Placebo (N = 747)	Tadalafil 5mg (N = 752)
**Demographics**		
Age group		
≥65, n (%)	306 (41.0)	308 (41.0)
≥75, n (%)	74 (9.9)	68 (9.0)
Ethnicity, n (%)		
White	650 (87.0)	649 (86.3)
American Indian or Alaska Native	49 (6.6)	49 (6.5)
Black or African American	16 (2.1)	17 (2.3)
BMI ≥30, n (%)	232 (31.1)	173 (23.0)
Mean BMI (SD), kg/m2	28.4 (4.4)	27.8 (4.0)
Alcohol intake, n (%)	465 (62.3)	457 (60.9)
Mean alcohol frequency (drinks per week), (SD)	6.02 (5.43)	5.84 (5.64)
Smoker, n (%)	99 (13.3)	90 (12.0)
**LUTS-BPH history**		
Mean IPSS total score (SD)	17.39 (5.95)	17.53 (5.73)
Mean IPSS voiding obstructive subscore (SD)	9.95 (4.09)	10.14 (3.97)
Mean IPSS storage irritative subscore (SD)	7.44 (2.96)	7.39 (2.92)
Mean IPSS QoL (SD)	3.66 (1.26)	3.60 (1.27)
Mean BII total score (SD)	5.24 (3.12)	5.09 (3.02)
Mean PGISS (SD)	4.04 (1.0)	3.99 (0.97)
LUTS severity, n (%)		
Mild-moderate (IPSS ≤20)	487 (65.2)	488 (64.9)
Severe (IPSS >20)	260 (34.8)	264 (35.1)
Mean Q_max_ (SD) ml/s	10.14 (4.23)	10.21 (3.63)
Previous LUTS therapies (within 12 months of screening), n (%)		
α-blockers	205 (27.4)	204 (27.1)
Therapy for overactive bladder, n (%)	7 (1.3)	10 (1.9)
**ED history**		
ED at baseline, n (%)	574 (76.9)	585 (77.8)
Mild ED (IIEF score 17–30), n (%)	147/574 (25.6)	148/585 (25.3)
Moderate ED (IIEF score 11–16), n (%)	340/574 (59.2)	339/585 (57.9)
Severe ED (IIEF score 1–10), n (%)	87/574 (15.2)	98/585 (16.8)
ED duration		
<3 months, n (%)	5 (0.9)	5 (0.9)
3–<6 months, n (%)	15 (2.6)	16 (2.7)
6–<12 months, n (%)	57 (9.9)	61 (10.4)
≥12 months, n (%)	497 (86.6)	503 (86.0)
Etiology of ED		
Organic	206 (27.6)	195 (25.9)
Psychogenic	26 (3.5)	25 (3.3)
Mixed	225 (30.1)	258 (34.3)
Unknown	117 (15.7)	107 (14.2)
International Index of Erectile Function (IIEF)		
Mean IIEF-EF (SD)	17.85 (8.48)	17.52 (8.51)
Mean IIEF-IS (SD)	7.89 (3.74)	7.89 (3.79)
Mean IIEF-OF (SD)	7.03 (3.17)	6.60 (3.26)
Mean IIEF-OS (SD)	5.79 (2.51)	5.77 (2.49)
Mean IIEF-SD (SD)	6.55 (1.89)	6.35 (1.88)
Previous ED therapies (within 12 months of screening), n (%)		
PDE-5 inhibitor, n (%)	167 (22.4)	173 (23.0)
**Laboratory parameters**		
Mean PSA (SD), ng/ml	1.74 (1.51)	1.87 (1.46)
Mean total testosterone (SD), ng/dl	3.76 (1.36)	3.66 (1.23)
Mean free testosterone (SD), ng/dl	0.06 (0.02)	0.06 (0.02)
Mean bioavailable testosterone (SD), ng/dl	1.40 (0.46)	1.38 (0.42)
Mean % bioavailable testosterone (SD)	39.26 (12.07)	39.70 (11.09)
Mean albumin (SD), g/dl	4.22 (0.33)	4.21 (0.32)
Mean sex hormone binding globulin (SHBG) (SD) nmol/l	40.37 (21.21)	41.02 (20.82)
**Comorbidities**		
Diabetes mellitus, n (%)	94 (12.6)	98 (13.0)
Hypertension, n (%)	288 (38.6)	300 (39.9)
Hyperlipidaemia, n (%)	152 (20.3)	183 (24.3)
Vasculitis, n (%)	0 (0)	3 (0.4)
Ischaemic heart disease, n (%)	44 (5.9)	43 (5.7)
Myocardial infarction, n (%)	11 (1.5)	11 (1.5)
Cardiac arrhythmia, n (%)	41 (5.5)	32 (4.3)
Cardiac failure, n (%)	6 (0.8)	5 (0.7)
Cardiomyopathy, n (%)	0 (0)	1 (0.1)
Other cardiac disorders, n (%)	19 (2.5)	25 (3.3)
Any cardiac disorder, n (%)	97 (13.0)	91 (12.1)
Haemorrhagic cerebrovascular disorders, n (%)	7 (0.9)	5 (0.7)
Ischaemic cerebrovascular disorders, n (%)	11 (1.5)	10 (1.3)
Other cerebrovascular disorders, n (%)	1 (0.1)	0 (0)
Any cerebrovascular disorder, n (%)	11 (1.5)	10 (1.3)
Peripheral vascular thrombosis or embolism, n (%)	18 (2.4)	17 (2.3)
Miscellaneous vascular disorder, n (%)	20 (2.7)	13 (1.7)
Other vascular disorder, n (%)	309 (41.4)	311 (41.4)
Cardiovascular disorder, n (%)	342 (45.8)	344 (45.7)
Renovascular disorder, n (%)	0 (0)	0 (0)
Renal impairment Glomerular filtration rate (GFR) ml/min/1.73m^2^		
Renal impairment stage I (GFR ≥90) n (%)	199 (26.8)	228 (30.6)
Renal impairment stage II (GFR 60–89) n (%)	327 (44.0)	368 (49.3)
**Concomitant medications**		
Testosterone, n (%)	1 (0.1)	1 (0.1)
Mean number anti-hypertensive medications*	0.90 (1.13)	0.92 (1.07)
α-blockers, n (%)	202 (27.0)	206 (27.4)
ß-blockers, n (%)	92 (12.3)	114 (15.2)
Calcium channel blockers, n (%)	76 (10.2)	68 (9.0)
Angiotensin converting enzyme inhibitors, n (%)	125 (16.7)	146 (19.4)
Angiotensin receptor blockers, n (%)	90 (12.0)	80 (10.6
Diuretics, n (%)	76 (10.2)	67 (8.9)
Centrally-acting sympathomimetics, n (%)	7 (0.9)	5 (0.7)
Other anti-hypertensive drugs, n (%)	2 (0.3)	6 (0.8)
Selective serotonin reuptake inhibitors, n (%)	11 (1.5)	6 (0.8)
Serotonin-norepinephrine reuptake inhibitors, n (%)	1 (0.1)	3 (0.4)
Antidepressants, n (%)	20 (2.7)	21 (2.8)
Tricyclic antidepressants, n (%)	1 (0.1)	1 (0.1)
Monoamine oxidase inhibitors, n (%)	0 (0)	0 (0)
Antipsychotics, n (%)	5 (0.7)	3 (0.4)
**Lipid-lowering therapies**		
Statins, n (%)	126 (16.9)	156 (20.7)
Other lipid-lowering drugs, n (%)	28 (3.7)	32 (4.3)
**Anti-diabetic medications**		
Sulfonylureas, n (%)	28 (3.7)	36 (4.8)
Alpha-glucosidase Inhibitors, n (%)	0 (0)	1 (0.1)
Amylin analogues, n (%)	0 (0)	0 (0)
Incretin mimetics, n (%)	0 (0)	2 (0.3
Dipeptidyl peptidase 4 inhibitors, n (%)	2 (0.3)	6 (0.8)
Biguanides, n (%)	58 (7.8)	66 (8.8)
Meglitinides, n (%)	2 (0.3)	1 (0.1)
Thiazlidinediones, n (%)	8 (1.1)	10 (1.3)
CYP3A4 Inhibitors, n (%)	88 (11.8)	101 (13.4)
**Treatment compliance**		
Hypertension treatment phase, n (%)	285 (38.2)	309 (41.1)
Oral agent, n (%)	70 (9.4)	79 (10.5)
Oral agent treatment phase, n (%)	71 (9.5)	79 (10.5)

*Eight derived variables defined in the SAP (Cluster cardiovascular drugs, cluster cerebrovascular drugs, cluster cardiovascular diseases, cluster cerebrovascular diseases, cluster anti-hypertensive drugs, cluster anti-psychotic drugs, cluster anti-diabetic drugs, cluster lipid-lowering drugs) were included in each assessment, which yields 106 variables. The inclusion of TRT as a variable, which is implicit in our evaluation, gives 107 variables in total.

Percent missing values were as follows: Testosterone (baseline), Free testosterone [18], % free testosterone, Bioavailable testosterone, % bioavailable testosterone = 79%; PSA (baseline) = 70%; Alcohol frequency = 38%; SHBG (baseline) = 33%; PGISS baseline, Previous overactive bladder therapy (Y/N) = 28%; Qmax, ED duration, ED aetiology, ED severity = 23%; IIEF baseline severity, IIEF-EF (baseline), IIEF-OF (baseline), IIEF-OS (baseline), IIEF-SD (baseline), IIEF-IS (baseline) = 14%; Albumin (baseline) = 2%; Renal impairment I, Renal impairment II, Renal impairment I (≥80), Renal impairment II (≥90) = 1%.

* prior α-blocking agents, β-blockers, calcium channels blockers, angiotensin converting enzyme inhibitors, angiotensin receptor blockers and diuretics.

IPSS, IPSS QoL, and BPH Impact Index (BII) were assessed in each of the four studies at baseline (after the 4 week placebo lead-in period following randomization) and after 12 weeks treatment (primary endpoint). Patient Global Impression of Improvement (PGI-I) was evaluated at baseline and endpoint in three of the four studies so as to assess the impression of change in urinary symptoms [6; 7; 8].

Overall, 107 baseline characteristics were included in the clinical data mining analysis (Table 1). Baseline characteristics were categorized as key or supportive and selected on the basis of clinical input from study authors that was derived from knowledge of the published literature and clinical experience. All IPSS, IPSS QoL and BII baseline scores and their subscores were key characteristics, in addition to age (<65 or ≥65 years), previous LUTS therapy and a history of ED (Table 1). Key characteristics were expected to be predictive for a response to treatment. Two primary and 6 secondary definitions of response were used (Table 2). The primary responder definitions were considered of equal importance and both were based on Minimal Clinically Important Differences (‘overall’ or ‘severity MCID’), a concept validated using an anchor-based approach [19]. MCID is a threshold that represents a CMI in patients’ health-related QoL as perceived by the patient [24]. ‘Overall MCID’ was defined as an improvement in IPSS total score of ≥3 for all patients (overall response) and ‘severity MCID’ defined as an improvement in IPSS total score of ≥2 for patients with mild-to-moderate LUTS and of 6 for those with severe LUTS [14; 19]. Secondary definitions of response were ranked in order of decreasing validation, although to the best of our knowledge they have not been subject to formal validation.

**Table 2:** Definition of treatment response on the IPSS, BII and PGI-I after 12 weeks treatment with tadalafil or placebo as used in the clinical data mining analysis.

**Instruments**
**Primary objectives**
**IPSS**
Reduction of ≥3 points in overall IPSS score [19; 20]
Improvement of ≥2 points in patients with IPSS baseline score <20 and of ≥6 points in patients with baseline score ≥20 [19]
**Secondary Objectives**
**IPSS QoL**
Reduction of ≥1 point in IPSS QoL [21]
**IPSS**
≥25% improvement in IPSS [22]
IPSS total score <12 in patients with prior score of ≥12
**BII**
Total score of <9
Reduction of >1 point [19]
**PGI-I**
Any improvement from baseline [23]

BII, BPH Impact Index; IPSS, International Prostate Symptom Score; PGI-I, Patient Global Impression of Improvement; QoL, quality of life.

Consistent with best practice, missing values were documented and data were screened for outliers [17]. Because of minor differences in study design, data were not necessarily available on all baseline variables. Outliers were defined as data points outside the range of [*Q*1–1.5 x *IQR*, *Q*3 + 1.5 x *IQR*], where Q1 and Q3 were the first and third quartiles and IQR the inter-quartile range, respectively. No imputation was conducted for missing values.

### Implementation

Bias stemming from the desire to achieve 100% prediction accuracy was controlled by following the pre-specified SAP as described earlier, which was approved by all study authors and peer reviewed by Lilly data mining experts prior to programming. A non-clinical benchmark data mining dataset was used for program development. Results from the clinical dataset were produced after program peer review, which was carried out by an independent statistician. All modifications of the analysis after this run were reported as post-hoc.

LR and DT models were selected as our data mining models as both can be presented visually and translated into easy decision rules or scores for practical use in medical applications [25; 26; 27] (S1 Technical Appendix). To avoid bias from an overly complex prediction model when a simple one would suffice [17], we compared all models against SDRs. These were implemented using the DT algorithm that was allowed to generate a single decision. In addition, SVM [28] (S2 Technical Appendix) and RF classifiers [29] were applied to obtain estimates for best prediction accuracy (S3 Technical Appendix).

**Fig 2:**
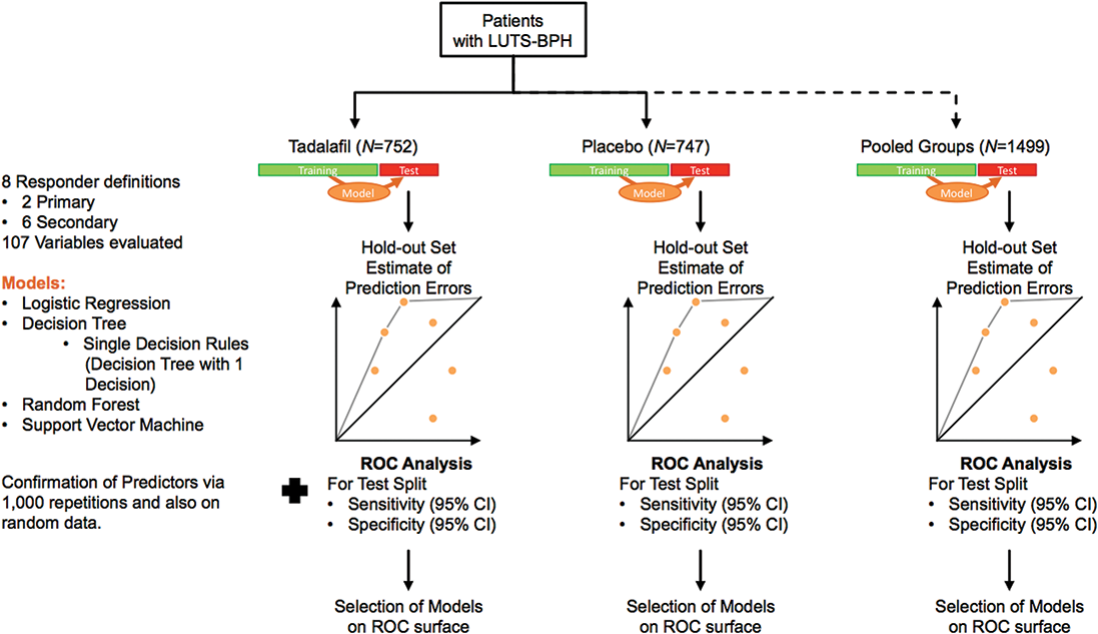
Data Analysis Flow.

The split set evaluation method was used to estimate prediction accuracy on unknown data. To this end, the database was randomly split into training (60% of the database) and test (40% of the database) subsets (Fig 2). Then LR, DT, SVM, RF and SDR models were generated on the training subset and used to predict the response of patients in the held-out test subset. Prediction models were generated for the tadalafil 5mg once daily and placebo groups. Prediction accuracy was measured by sensitivity (true positives) and specificity (true negatives), for which 95% confidence intervals were calculated. Sensitivity and specificity were calculated as follows:

$$\text{Sensitivity}\;=\;\frac{\text{TP}}{\text{TP}\;+\;\text{FP}};\text{Specificity}\;=\;\frac{\text{TN}}{\text{TN}\;+\;\text{FN}}$$

In the equation, TP and TN denote the true positive and true negative predictions and FP and FN denote the false positive and false negative predictions on the test split.

Receiver Operating Curve (ROC) analysis was used to identify optimal prediction models lying on the ROC surface [30] (Fig 2). For ROC curve interpretation we adopted a systematic approach in which models on the ROC surface were first documented by their respective sensitivity and specificity, after which the model on the ROC surface that gave equal weight to false positive and false negative errors was discussed in detail. For the primary objectives, the resulting sensitivity and specificity was then compared to the Q1–Q3 range of 1,000 repeated runs of the 60:40 split set evaluation to ensure consistency (non-random data) (S4 Technical Appendix). Additionally, these results were compared with results obtained from 1,000 repeated runs with a randomly permuted response variable (random data). Finally, sensitivity and specificity findings were compared against an 80% cut-off, representing a performance threshold suitable for routine clinical use.

All analyses were programmed in R 2.15.1 using packages rpart (3.1–53; for DTs) [31] e1017 (1.6–1; for SVM) [32] and Random Forest (4.6–7; for RFs) [33].

Post-hoc sensitivity analyses were conducted to determine whether or not excluding a minimised combination of characteristics affected primarily by missing data would allow the generation of improved models (S5 Technical Appendix). Again, emphasis was placed on those models being optimal when false positive and false negative errors were of equal importance (i.e. a sensitivity and specificity threshold of >80%).

## Results

### Overall findings

Analyses were based on pooled data from four randomized, placebo-controlled trials that primarily compared the effect of 12 weeks treatment with tadalafil 5mg once daily with placebo on symptomatic LUTS improvement in men with LUTS-BPH. Baseline characteristics of patients in the two treatment groups were well balanced (Table 1). There was negligible heterogeneity across the four studies.

The complete ITT population was used in all our models. However, depending on the algorithm, there were exclusions due to missing response or incomplete data from the run. LR, SVM and RF implementation could not be used with incomplete patient records, whereas DTs were able to handle missing predictor, but not missing response variable information, by using ‘surrogate splits’, for which we allowed 5. Post-hoc sensitivity analysis was used to explore the influence of missing data on the primary result. The set of predictors was reduced such that a sufficient number of complete records were available to the logistic regression, SVM and RF training algorithms. In the end, all patients included in the ITT population were available for inclusion in the data mining algorithms and no patient was excluded for reasons other than technical ones.

Based on these data, the output from our clinical data mining analysis did not find sufficiently good predictors of treatment response to placebo or tadalafil. None of the 107 pre-selected baseline characteristics achieved a combined sensitivity and specificity of >80% that would enable reliable allocation of an individual patient to either the tadalafil responder or non-responder group.

As the detailed results presented below demonstrate, IPSS baseline (mild/moderate vs. severe group) for both placebo and tadalafil 5mg once daily was found several times on the ROC surface and generated the highest combined sensitivity and specificity of 70% and ~50%, respectively, for all analyses.

### Significance of outliers

Outliers were assessed in this clinical data mining study but were not removed for the reasons described earlier. The assessment of outliers led to relatively few observations. It is worth noting that 3 baseline characteristics had skewed distributions. These were maximum urinary flow rate (Q_max_), body mass index (BMI), and frequency of alcohol intake, all of which had >23 outliers in the upper range of their respective scales. Full outlier results are given in the accompanying Supporting Information (S6 Technical Appendix).

### Primary Objectives

In our ROC curve analyses, models on the ROC surface represented an optimal trade-off between prediction errors (false positive vs. false negative predictions). Here we describe results from the model in which we observed an equal trade-off between both errors as determined by ROC curve analysis. Only SDR models were obtained for the pre-specified analyses predicting ‘severity MCID’ and ‘overall MCID’ response. A reduction of ≥3 points in overall IPSS score, or improvement of ≥2 points in patients with IPSS baseline score <20 and of ≥6 points in patients with baseline score ≥20 were the primary objectives.

Prediction of ‘severity MCID’ response in the tadalafil 5mg once daily group produced SDR models on the ROC surface for IPSS severity group (mild/moderate vs. severe) and IPSS voiding subscore only (Table 3). The model with equal importance for FP and FN error was based on IPSS severity group. These results (using this model) were supported by repeat evaluations, which lay within the Q1–Q3 ranges for sensitivity and specificity of 68–72% and 45–50%, respectively. Q1–Q3 ranges for random data were 34–66% for sensitivity, and as such did not overlap with the runs on non-random data, and 34–66% for specificity. For subjects in the mild/moderate group, this model predicted a positive ‘severity MCID’ response.

**Table 3:** Primary results for both treatment groups.

**Severity MCID**		
Treatment group	Sensitivity % (95% CI)	Specificity % (95% CI)
**Tadalafil 5mg**		
IPSS baseline (mild/moderate vs. severe)*	70 (63, 76)	50 (40, 60)
IPSS voiding/obstructive subscore	97 (94, 99)	7 (3, 15)
**Placebo**		
Bioavailable testosterone	13 (8, 19)	91 (85, 95)
ED aetiology	49 (41, 57)	61 (52, 69)
IPSS baseline (mild/moderate vs. severe)*	74 (66, 80)	39 (30, 47)
Cluster lipid-lowering medications	88 (82, 93)	23 (16, 31)
Antidepressants (Y/N)	98 (94, 100)	5 (2, 10)
5-α-reductase inhibitors (Y/N)	99 (96, 100)	2 (0, 6)
**Overall MCID**		
**Tadalafil 5mg**		
Ethnicity	9 (5,14)	98 (92, 100)
IPSS baseline (mild/moderate vs. severe)	38 (32, 45)	71 (61, 80)
IPSS voiding obstructive subscore*	96 (92, 98)	20 (12, 29)
**Placebo**		
Cluster antidiabetic drugs	11 (6, 16)	95 (89, 98)
IPSS baseline (mild/moderate vs. severe)	40 (33, 48)	73 (65, 81)
Alcohol use (Y/N)	45 (37, 53)	70 (61, 77)
IPSS voiding/obstructive subscore*	94 (89, 97)	23 (16, 31)

*Model on ROC surface with best performance if false positive and false negative errors are equally important.

CI, confidence interval; ED, erectile dysfunction; IPSS, International Prostate Symptom Score; MCID, Minimal Clinically Important Differences; N, no; PSA, prostate specific antigen; ROC, Receiver Operating Curve; Y, yes.

‘Severity MCID’ response in the placebo group was predicted by six SDR models lying on the ROC surface that included bioavailable testosterone, ED etiology, IPSS severity, cluster of lipid-lowering medications, antidepressants, and use of 5-α-reductase inhibitors (Table 3). Again, IPSS severity achieved the combination of best sensitivity and specificity when positive and negative prediction errors were of equal importance. The Q1–Q3 range for all evaluations was 71–74% for sensitivity and 39–44% for specificity, while random data yielded sensitivities of 32–65% and specificities of 36–68%. Again, there was no overlap with evaluations on non-random data, increasing confidence that the effect was not simply due to random effect. This model also predicted a positive ‘severity MCID’ response for subjects in the mild/moderate group.

SDR models predicting ‘overall MCID’ response in the tadalafil 5mg once daily group were based on ethnicity, IPSS severity, and IPSS voiding subscores (Table 3). Here, the IPSS voiding subscore SDR model achieved optimal predictions when false positive errors were assumed to have the same importance as false negative errors. Q1–Q3 ranges were 77–96% for sensitivity and 13–29% for specificity. For random data these were 8–89% for sensitivity, respectively, and 11–91% for specificity, respectively.

‘Overall MCID’ for the placebo group was best predicted by SDR models that included cluster of anti-diabetic drugs, IPSS severity, alcohol usage, and IPSS voiding subscore (Table 3). Giving equal importance to false positives and to false negatives, IPSS voiding scores obtained the best predictions. Subjects with an IPSS voiding subscore >5.5 were predicted to have a higher likelihood of ‘overall MCID’ response. Q1–Q3 ranges for this model in all evaluations were 93–95% for sensitivities and 19–23% for specificities. The corresponding results for random data were 10–88% for sensitivities and 13–90% for specificities.

The IPSS severity categories (mild/moderate vs. severe) based on a cut-off of 20 were part of the ROC surface regardless of MCID definition and regardless of treatment group (i.e. tadalafil 5mg once daily or placebo). IPSS voiding subscore was found on the ROC surface for ‘overall MCID’ prediction.

### Secondary Objectives

Estimates for sensitivities and specificities for each of the secondary objectives for the two treatment groups are presented in Tables 4 and 5. The SDR models achieving optimal prediction performance when false positive predictions are given the same importance as false negative predictions are marked with a star (*) and are the results on which we have focused.

**Table 4:** Secondary Results (Tadalafil 5mg once daily).

**Variable Description**	**Sensitivity % (95% CI)**	**Specificity % (95% CI)**
**IPSS QoL improvement**		
Myocardial infarction (Y/N)	2 (0, 5)	100 (97, 100)
Smoking (Y/N)	15 (10, 21)	94 (87, 97)
Number of anti-hypertensive medications*	48 (41, 56)	66 (56, 75)
Cardiovascular disorders cluster	57 (49, 64)	58 (48, 68)
Statins (Y/N)	79 (73, 85)	33 (25, 43)
IPSS QoL (baseline)	98 (95, 99)	11 (6, 19)
Antidepressants (Y/N)	99 (96, 100)	6 (3, 13)
**IPSS 25% change**		
Q_max_	10 (6,16)	94 (89, 98)
IIEF baseline severity	19 (14, 26)	89 (82, 94)
Renal impairment II	52 (44, 59)	60 (50, 68)
Hypertension treatment phase (Y/N)*	66 (59, 74)	46 (37, 55)
Hyperlipidemia (Y/N)	81 (74, 87)	29 (22, 38)
IPSS voiding obstructive subscore (baseline)	95 (91, 98)	13 (7, 20)
Cluster antidepressant-antipsychotic	99 (96, 100)	5 (2, 10)
Renal impairment I	100 (98, NaN)	2 (0, 6)
**IPSS Score <12**		
IPSS total score (baseline)*	60 (52, 68)	78 (70, 85)
IPSS baseline group	80 (73, 86)	58 (50, 67)
Previous overactive bladder therapy (Y/N)	100 (98, NaN)	2 (0, 6)
**BII Score < 9**		
Ethnicity	7 (4,10)	100 (79, 100)
IPSS QoL (baseline)	51 (45, 57)	88 (62, 98)
BII total score (baseline)*	68 (62, 73)	75 (48, 93)
Antidepressants (Y/N)	98 (95, 99)	6 (0, 30)
**BII improvement**		
Number of anti-hypertensive medications**	2 (1, 6)	99 (95, 100)
BII total score (baseline)*	97 (93, 99)	25 (17, 34)
**PGI-I**		
% bioavailable testosterone*	62 (24, 91)	84 (78, 89)
Cluster anti-hypertensive drugs	88 (47, 100)	44 (37, 52)
Albumin (baseline)	100 (63, NaN)	17 (12, 23)

*Model on ROC surface with best performance if false positive and false negative errors are equally important.

** Alpha-Blockers, beta-blockers, calcium channels blockers, angiotensin converting enzyme inhibitors, angiotensin receptor blockers and diuretics.

BII, BPH Impact Index; CI, confidence interval; IIEF, International Index of Erectile Function; IPSS, International Prostate Symptom Score; N, no; PGI, Patient Global Impression of Improvement; Q_max_, maximal flow rate; QoL, quality of life; ROC, Receiver Operating Curve; Y, yes.

**Table 5:** Secondary Results (Placebo).

Variable Description	Sensitivity % (95% CI)	Specificity % (95% CI)
**IPSS QoL improvement**		
Free testosterone	7 (4, 13)	97 (93, 99)
ED duration	16 (11, 23)	91 (85, 95)
ED etiology*	37 (29, 45)	76 (69, 83)
IPSS QoL (baseline)	89 (83, 93)	22 (16, 30)
Number of anti-hypertensive medications **	99 (96, 100)	3 (1, 7)
**IPSS 25% Change**		
ED duration	4 (1, 9)	100 (98, 100)
Ethnicity	10 (6, 17)	96 (92, 98)
Cluster anti-hypertensive Drugs	52 (43, 61)	63 (55, 70)
PGI-S baseline*	69 (60, 77)	48 (41, 56)
IIEF-IS (baseline)	85 (77, 91)	29 (22, 36)
Other lipid lowering drugs (Y/N)	99 (96, 100)	6 (3, 11)
**IPSS Score <12**		
Ethnicity	13 (8, 20)	95 (91, 98)
IPSS storage irritative subscore (baseline)	58 (49, 67)	75 (68, 81)
IPSS total score (baseline)*	69 (60, 77)	68 (60, 75)
IPSS baseline group	81 (73, 88)	51 (43, 58)
Other lipid lowering drugs (Y/N)	100 (97, NaN)	4 (2, 8)
**BII Score <9**		
Ethnicity	8 (5, 11)	100 (85, 100)
IPSS baseline group*	72 (66, 77)	91 (71, 99)
BII total score (baseline)	89 (85, 93)	64 (41, 83)
**BII Improvement**		
Alcohol frequency	3 (1, 7)	98 (95, 100)
IPSS baseline group	45 (37, 53)	73 (64, 80)
BII total score (baseline)*	87 (81, 92)	41 (32, 50)
IPSS storage irritative subscore (baseline)	96 (92, 99)	16 (10, 23)
**PGI-I**		
Testosterone (baseline)	18 (2, 52)	92 (87, 96)
SHBG (baseline)*	91 (59, 100)	47 (39, 54)
Renal impairment I	100 (72, NaN)	32 (25, 39)

*Model on ROC surface with best performance if false positive and false negative errors are equally important.

** Alpha-Blockers, beta-blockers, calcium channels blockers, angiotensin converting enzyme inhibitors, angiotensin receptor blockers and diuretics.

BII, BPH Impact Index; CI, confidence interval; ED, erectile dysfunction; IIEF, International Index of Erectile Function; IPSS, International Prostate Symptom Score; N, no; PGI-I, Patient Global Impression of Improvement; PGISS, Patient Global Incontinence Severity Score; QoL, quality of life; ROC, Receiver Operating Curve; SHBG, sex hormone binding globulin; Y, yes.

A reduction of ≥1 point on the IPSS QoL question was the first secondary objective. SDR models found on the ROC surfaces included number of anti-hypertensive medications for the tadalafil 5mg once daily group, and ED etiology (mixed or psychogenic) for the placebo group to predict improvements.

A reduction in the IPSS total score of 25% from baseline to 12 weeks was the next secondary objective, and SDR models on the ROC surface included presence of hypertension during treatment for the tadalafil group and PGI-S (<5) at baseline for the placebo group.

Achieving an IPSS total score <12 points at 12 weeks was the third secondary objective. An IPSS score <12 was predicted using IPSS total score for the tadalafil 5mg once daily and placebo groups. Cut-off for response was selected as <16 for tadalafil 5mg once daily and placebo by SDR models on the ROC surface giving equal importance to false positive and false negative predictions.

A reduction to <9 points on the BII total score after 12 weeks treatment was the fourth secondary objective. IPSS severity (mild/moderate) was used to predict BII <9 after 12 weeks treatment for the placebo group, while the BII total score (<6.5) was used by the SDR predicting response/improvement in tadalafil-treated patients.

A reduction of >0.5 point on the BII scale was the fifth secondary objective. BII total score at baseline was used to predict any improvements in BII by the SDR models. The cut-offs employed were ≥1.5 and ≥2.5 for response in the tadalafil 5mg once daily and placebo groups, respectively.

The final secondary objective was any improvement on the PGI scale. SDR models lying on the ROC surface that gave equal importance to false positives and false negatives in predicting improvements were, % bioavailable testosterone (≥35%) for the tadalafil 5mg once daily group and sex hormone binding globulin (SHBG) (<42nmol/l) for the placebo group, respectively.

### Post-hoc Sensitivity Analysis

All pre-specified analyses returned only SDR models. LR, SVM, RF and DT approaches did not yield models because missing values, that included parameters that were either not measured or intended for collection, resulted in an insufficient number of complete patient records. Testosterone measurements were the key driver, responsible for 79% of incomplete records, while missing PSA assessments accounted for 70% of records, followed by frequency of alcohol intake and SHBG assessments (both missing in >30% of cases). Finally, PGI assessment (PGI-I was assessed in only 3 of the 4 studies), previous overactive bladder therapy, ED characteristics and assessment of Q_max_ were missing for 20% to 30% of patients.

Table 6 details sensitivities and specificities on held-out test data from non-SDR models lying on the ROC surface when testosterone, alcohol intake, Q_max_, SHBG, albumin, PGI-S and PSA were excluded. For 13 of these models, pre-selection via a t-test filter improved prediction performance (S7 Technical Appendix). In these cases the pre-selected variables are given in the last column of the table. Only 4 of the models were RF; not a single SVM was observed. Of the better performing models, sensitivity and specificity were best with respect to BII total score of <9. DTs for the tadalafil 5mg once daily group achieved a sensitivity of 77% (95% CI: 0.72, 0.82) and specificity of 62% (95% CI: 0.35, 0.85).

**Table 6:** Exploratory Results.

Groups	Model	Sensitivity % (95% CI)	Specificity % (95% CI)	Variables included, if based on feature selection
**Severity MCID**				
Placebo	DT	58 (50, 66)	56 (47, 65)	
**Overall MCID**				
Tadalafil	DT	67 (60, 74)	51 (40, 61)	IPSS total and storage score
Placebo	DT	92 (87, 96)	25 (18, 33)	IPSS total and voiding score
**QoL Improvement**				
Tadalafil	DT	88 (83, 92)	31 (23, 41)	IPSS QoL score
Tadalafil	DT	58 (51, 65)	63 (53, 72)	
Placebo	RF	61 (53, 69)	55 (46, 63)	IPSS QoL score
Placebo	DT	89 (83, 93)	22 (16, 30)	IPSS QoL score
**IPSS 25%**				
**BII <9**				
Tadalafil	RF	99 (97, 100)	6 (0, 30)	BII total score, IPSS total, voiding and storage, IPSS QoL score
Tadalafil	DT	77 (72, 82)	62 (35, 85)	BII total score, IPSS total, voiding and storage, IPSS QoL score
Placebo	RF	98 (96, 99)	23 (8, 45)	Number of anti-hypertensive treatments, Study treatment compliance, BII total score baseline, IPSS storage,voiding and total score, Cardiovascular disorders cluster, Cluster cardiovascular disorders (CLUSTCARDVDIS), Cluster anti-diabetic drugs
**BII Improvement**				
Tadalafil	RF	65 (57, 72)	60 (50, 69)	BII total score
Placebo	DT	72 (64, 79)	58 (49, 67)	BII and IPSS total score, IPSS voiding score
**PGI Improvement**				
Placebo	DT	64 (31, 89)	69 (61, 75)	

Models were generated on dataset excluding testosterone, alcohol frequency, Qmax, SHBG, Albumin, PGI, and PSA

BII, BPH Impact Index; CI, confidence interval; DT, Decision Tree; IPSS, International Prostate Symptom Score; MCID, Minimally Clinically Important Differences; PGI-I, Patient Global Impression of Improvement; PSA, prostate specific antigen; Q_max_, maximal flow rate; QoL, quality of life; RF, Random Forest; SHBG, sex hormone binding globulin.

## Discussion

Identifying predictors of response to drug therapy can be beneficial, especially where significant improvements in patient health-related QoL are sought, such as in LUTS-BPH where symptom relief is the primary goal of treatment for the majority of men. It also has benefits in an era where patients are encouraged to take an active role in treatment decisions alongside their physician.

The objective of this clinical data mining study was to identify prediction models and associated patient baseline characteristics that could be used in clinical practice to predict treatment response to tadalafil 5mg once daily among patients with a diagnosis of LUTS-BPH. To the best of our knowledge, this is the first clinical data mining analysis to use mathematical modelling in studies of patients with LUTS-BPH.

To meet this objective, we adopted a rigorous data mining approach involving commonly used models and evaluated their discriminative ability on held-out data using eight different measures of treatment response and 107 possible predictors. These were chosen from a large patient population enrolled in a series of almost identical, placebo-controlled, randomized studies of the same duration of randomized treatment and with similar inclusion/exclusion criteria. Results were backed up by repeated evaluations and comparison to non-informative data to control for bias.

As our results have demonstrated, we did not to obtain any sensitivities or specificities above an 80% threshold for the specified baseline characteristics. In other words, at this threshold there would be a 20% risk of an incorrect prediction, which we would argue is an acceptable basis on which to predict treatment response in a non-malignant condition in clinical practice. Thus, using our data from four clinical trials and modelling methods, no single predictive rule emerged from which a treatment algorithm could be developed to clinically guide the use of tadalafil 5mg once daily in patients with LUTS-BPH. Similarly, we found no characteristics that determined response to LUTS-BPH treatment when placebo is used. These findings applied to both primary and secondary objectives.

Across the 107 baseline characteristics, there was evidence that with respect to ‘severity MCID’, LUTS severity at baseline as measured by IPSS score (mild-moderate ≤20 vs. severe >20) had sensitivity and specificity levels that approached 70% and 50%, respectively. While this level of prediction is marginally better than random guessing, it is still too low for clinical use. However, IPSS continues to underpin assessments with respect to baseline symptom severity and monitoring symptom progression in cases of “watchful waiting” [34]. This may be due to the fact that during its validation, care was taken to generate a predictive questionnaire [10; 21].

Several analyses of pooled data from the four clinical trials of tadalafil versus placebo that were used in this clinical data mining study have shown that tadalafil significantly improves symptoms of LUTS-BPH, including small but significant improvements in Q_max_ [35] with concomitant improvements in QoL [11; 36]. Subsequent analyses revealed improvement in both IPSS storage and voiding subscores [12], and that improvements in LUTS occurred irrespective of the presence of co-existing ED [37]. Thus, tadalafil has therapeutic benefit beyond its effects on ED in men with comorbid LUTS-BPH. These findings have been confirmed in a prospective, naturalistic observational study (TadaLutsEd), which closely mirrors routine clinical practice. In this non-selective study, 86% of men aged 50 years and older with LUTS-BPH saw an improvement in urinary symptoms following 6 weeks treatment with tadalafil 5mg once daily [38].

A subgroup analysis of the effects of tadalafil in various patient subgroups concluded that tadalafil improves LUTS-BPH symptoms, as measured by the IPSS, across all clinical subgroups that included LUTS severity (IPSS ≤20/>20) and previous use of α-blocking agents [13]. However, while this analysis looked at the various subgroups from a population perspective and, as such, evaluates improvement *on average*, our work crucially looks at it from the perspective of the physician and the individual patient (i.e. predicting the improvement *on an individual basis*). Both analyses are consistent in that efficacy occurred across all subgroups in the pooled analysis of data from the four clinical trials, while no reliable predictor of response was found in our analysis of the same trials on an individual patient basis.

Given that tadalafil provides early symptomatic relief [6] across a wide range of men with LUTS-BPH, including those with ED and other significant comorbidities, it is perhaps not surprising that we were unable to identify individual predictors of response to placebo or tadalafil 5mg once daily despite rigorous data mining. Many examples exist in the literature of predictors of response (or failure to respond) to drug therapy that include the use of drugs for LUTS-BPH. For example, large prostate volume and more severe symptoms at baseline have been identified as predictive factors for failure to respond to first-line medical therapy for LUTS-BPH [39]. Severity of symptoms is a strong influence on the extent to which patients judge treatment to give clinically meaningful improvement [19]–greater severity requires a proportionately greater improvement in symptom relief for patients to perceive the same degree of improvement as those with less severe disease [16]. A systematic review and meta-analysis of the use of PDE-5 inhibitors in LUTS-BPH suggested that younger men with lower BMI and severe urinary symptoms were the best candidates for PDE-5 inhibitor therapy [40], a finding we were unable to demonstrate and confirm in our analysis when examining patients treated with tadalafil or using placebo.

We did, however, identify some potential candidates for predicting treatment response. In addition to IPSS-related characteristics, we found that bioavailable testosterone, ED etiology, cluster of lipid-lowering medications, antidepressants and previous use of 5-α-reductase inhibitors may have potential as predictors for treatment response, especially in relation to ‘severity MCID’ response. Although substantial further work is needed to test these observations, there is some independent evidence to suggest that some, if not all, may be viable candidates. A recent study on the effects of tadalafil 5mg in men with hypogonadism and LUTS-BPH showed that while tadalafil was effective in both men with and without hypogonadism, IPSS storage subscore and IPSS-QoL was appreciably greater in men without hypogonadism than those with low testosterone levels [41]. There is also evidence to suggest that depression, anxiety and somatization may influence the clinical manifestation of LUTS-BPH and that anxious patients respond less well to treatment [42]. Conceivably, treatment with antidepressants could play in role in not only alleviating symptoms of depression and anxiety but also increasing the likelihood of response to specific LUTS-BPH therapy, something for which there is now published evidence [43].

In this study we chose to use established models for prediction, such as LRs, DTs, SVMs and RFs, rather than newer and more complex models. Surprisingly, none of them showed robustness with regards to handling missing data. This was unexpected, especially for DTs and RFs. Current data mining research is focused on developing models that achieve ever better prediction methods (on complete datasets), while simultaneously ignoring the problem of missing information that could be informative but could also completely compromise the method. In our modelling study, even DTs that have an integrated mechanism for dealing with missing data via surrogate splits, often failed to achieve better performance over models that made only a single decision. Only nine DTs were found on the ROC surface and of these, six required pre-selection of variables via a t-test filter. This clearly highlights the importance of this issue in clinical data mining research.

Despite its strengths, which include a pre-specified program of statistical analyses, this study has several limitations. Firstly, there was no subsequent independent study to validate our results. It is also possible that we may not have collected the “true factor” for predicting response, even though we examined 107 baseline characteristics. Better methods could have been employed to fine tune model parameters, especially for the SVM. For example, a triple split set evaluation, consisting of a training split for model generation, a validation split for model selection, and test split for hold-out evaluation could have been used to fine tune the selection of better generalising models. Evaluation of the training-test set bias did not, however, indicate a need for such additional complexity. An alternative pre-filtering step could have been used, adding clustering information as supportive predictor information, adding de-noising (whitening) data pre-processing steps, or using statistical bootstrapping. With respect to SVM, we employed the radial basis kernel but we could have used further kernels, such as random walk kernels, optimal matching kernels, or other kernel types or kernel machines for training this algorithm on our data. There were also limitations inherent in the trial inclusion and exclusion criteria; for example, patients with post-void residual urine volume >300ml were excluded and prostate volume was not directly assessed, although PSA can be used as a surrogate for prostate size.

In conclusion, none of the approaches presented here led to a prediction model with sufficient accuracy for the development of a tailoring algorithm for tadalafil 5mg once daily or placebo in LUTS-BPH. Thus, the ideal patient profile for which tadalafil should be prescribed with respect to baseline demographics, medical history, IPSS, International Index of Erectile Function (IIEF) score and Q_max_ remains as yet unknown. Although the response to treatment in an *individual patient* cannot be reliably predicted from the characteristics and methods we have evaluated so far, this does not mean that patients with LUTS-BPH are not likely to respond *on average* to treatment with placebo or tadalafil 5mg once daily. Among the approximately two-thirds of men with LUTS-BPH who achieved CMI following treatment with tadalafil 5mg, over half achieved CMI after one week of therapy and over 70% within 4 weeks [44].

Although this study did not identify any pre-existing patient characteristics that might predict a treatment-response, tadalafil 5mg once daily has been shown to effectively impact LUTS-BPH across a range of patient subgroups. Therefore, the decision to treat an individual case of LUTS-BPH with tadalafil 5mg once daily continues to rest on medical assessment of the patient, consideration of contra-indications, presence of co-existing conditions, with the patient’s expectations and preferences leading to mutual patient-physician agreement. This approach is entirely compatible with the current concept of shared decision making, in which the patient’s voice should also be heard as an integral part of the treatment decision [45], especially for a condition in which part of the symptomatic improvement is a strong placebo response [16].

## Supporting Information

S1 Technical Appendix (DOCX)

S2 Technical Appendix (DOCX)

S3 Technical Appendix (DOCX)

S4 Technical Appendix (DOCX)

S5 Technical Appendix (DOCX)

S6 Technical Appendix (DOCX)

S7 Technical Appendix (DOCX)
