# Supplementary material for: Predictors of Individual Response to Placebo or Tadalafil 5mg among Men with Lower Urinary Tract Symptoms Secondary to Benign Prostatic Hyperplasia: An Integrated Clinical Data Mining Analysis
Source: PLoS One. 2015 Aug 18;10(8):e0135484. doi: 10.1371/journal.pone.0135484 (PMC4540425; doi:10.1371/journal.pone.0135484)
Supplement: S2 Technical Appendix — (DOCX) [file pone.0135484.s002.docx]

**“S2 Technical Appendix”**

Parameters for the C-classification SVM were selected via grid search for the Radial Basis Function kernel (cost ranging from 1 to 2^5^, Gamma ranging from 2^-8^ to 2^5^).
