# Supplementary material for: Predictors of Individual Response to Placebo or Tadalafil 5mg among Men with Lower Urinary Tract Symptoms Secondary to Benign Prostatic Hyperplasia: An Integrated Clinical Data Mining Analysis
Source: PLoS One. 2015 Aug 18;10(8):e0135484. doi: 10.1371/journal.pone.0135484 (PMC4540425; doi:10.1371/journal.pone.0135484)
Supplement: S3 Technical Appendix — (DOCX) [file pone.0135484.s003.docx]

**“S3 Technical Appendix”**

An imbalance between positives (responders) and negatives (non-responders) was expected. To avoid generating models on the training split that returned only a one-sided prediction, the situation was simulated as if the model was generated with an equal number of positive and negative cases using weighting. The weights were set such that the sum of all positive cases equalled the sum of all negative cases and the sum all weights equalled 1.
