# Supplementary material for: Predictors of Individual Response to Placebo or Tadalafil 5mg among Men with Lower Urinary Tract Symptoms Secondary to Benign Prostatic Hyperplasia: An Integrated Clinical Data Mining Analysis
Source: PLoS One. 2015 Aug 18;10(8):e0135484. doi: 10.1371/journal.pone.0135484 (PMC4540425; doi:10.1371/journal.pone.0135484)
Supplement: S4 Technical Appendix — (DOCX) [file pone.0135484.s004.docx]

**“S4 Technical Appendix”**

Results from the first seed were reported and compared against the distribution of results from all splits. To protect against induction bias [Mitchell, “Machine Learning”, 1997], that is bias stemming from using overly flexible data mining models, we repeated each split set evaluation with a randomly permuted response variable. Correct predictions on such a dataset are by pure chance and provide an estimate on what data mining can produce when no association is present. Since we also assessed the distribution of sensitivities and specificities for a non-permuted response variable, we were able to compare their distributions on our sensefull, clinical dataset vs this artificially created non-sense dataset, which has still the same overall patients characteristics. To this end, we looked at the IQR ranges for sensitivities and specificities, comprising 50% of all results, and checked whether they overlapped. In case, the IQR for the sensefull dataset did not overlap with the IQR for the non-sense dataset, we considered this as supportive for the presence of information in the data that can be learned well.

Mitchell TM (1997) Machine Learning. Pub. McGraw –Hill, Inc. New York, NY, USA.
