# Supplementary material for: Predictors of Individual Response to Placebo or Tadalafil 5mg among Men with Lower Urinary Tract Symptoms Secondary to Benign Prostatic Hyperplasia: An Integrated Clinical Data Mining Analysis
Source: PLoS One. 2015 Aug 18;10(8):e0135484. doi: 10.1371/journal.pone.0135484 (PMC4540425; doi:10.1371/journal.pone.0135484)
Supplement: S5 Technical Appendix — (DOCX) [file pone.0135484.s005.docx]

**“S5 Technical Appendix”**

As the LR, SVM and RF algorithms did not achieve acceptable prediction accuracy due to the structure of missing data we conducted a series of post-hoc sensitivity analyses excluding characteristics causing an insufficient number of complete observations. Here we report the sensitivity analysis excluding testosterone, alcohol frequency, Q_max_, SHBG, Albumin, PGI, and PSA at baseline.
