# Supplementary material for: Predictors of Individual Response to Placebo or Tadalafil 5mg among Men with Lower Urinary Tract Symptoms Secondary to Benign Prostatic Hyperplasia: An Integrated Clinical Data Mining Analysis
Source: PLoS One. 2015 Aug 18;10(8):e0135484. doi: 10.1371/journal.pone.0135484 (PMC4540425; doi:10.1371/journal.pone.0135484)
Supplement: S6 Technical Appendix — (DOCX) [file pone.0135484.s006.docx]

**“S6 Technical Appendix”**

In total 15 of the characteristics presented with more than 1 outlier above or beneath the cut-offs. One cluster of outliers related to several laboratory parameters. These included testosterone, PSA, albumin and SHBG, with 3 to 45 outliers on the upper range. Another set of outliers was mainly driven by numeric, e.g. when the Q3 was exactly the same as Q1, leading to an IQR of 0, and therefore the outlier cut-off boundaries collapsed with the Q1 and Q3. This happened for the PGI-S, where 50% of all patients reported a PGI-S at baseline of 4.0 leading to a Q1 and Q3 of 4 and the corresponding IQR, 0.0. With a maximum of 6 and a minimum of 1, this led to 257 patients lying beneath and 156 above 4, classified as outliers. Similarly, the number of anti-hypertensive drugs, where the IQR was 1.0; 75% of all patients took 1 (Q3) anti-hypertensive drug and 146 patients 2.5 or more anti-hypertensive drugs.
Most remarkable was the distribution of study treatment compliance (the proportion of pills taken versus pills scheduled), with 146 outliers beneath and 86 above the cut-offs.
