# Supplementary material for: Predictors of Individual Response to Placebo or Tadalafil 5mg among Men with Lower Urinary Tract Symptoms Secondary to Benign Prostatic Hyperplasia: An Integrated Clinical Data Mining Analysis
Source: PLoS One. 2015 Aug 18;10(8):e0135484. doi: 10.1371/journal.pone.0135484 (PMC4540425; doi:10.1371/journal.pone.0135484)
Supplement: S7 Technical Appendix — (DOCX) [file pone.0135484.s007.docx]

**“S7 Technical Appendix”**

The accuracy of prediction algorithms can be hampered if too many characteristics are included. This variable selection problem and its associated variable selection bias can be mitigated by using a filter approach. Each algorithm was therefore run additionally with a pre-selected set of characteristics. The selection was created by applying a two-sample t-test between responder and non-responder groups for each characteristic, controlling the p-values for the false discovery rate (FDR) using the Benjamini-Hochberg method [Benjamini & Hochberg, 1995] and choosing only those characteristics that were significant at the 5% level. In addition, the logistic regression was also run post-hoc with forward-, backward- and stepwise selection procedures.

Benjamini Y, Hochberg Y (1995) Controlling for false discovery rate: A practical and powerful approach to multiple testing. J Royal Statistical Society 57: 289–300.
